# Echocardiographic Screening of Anomalous Origin of Coronary Arteries in Athletes with a Focus on High Take-Off

**DOI:** 10.3390/healthcare9020231

**Published:** 2021-02-20

**Authors:** Massimiliano Cantinotti, Raffaele Giordano, Nadia Assanta, Martin Koestenberger, Eliana Franchi, Pietro Marchese, Alberto Clemente, Shelby Kutty, Flavio D’Ascenzi

**Affiliations:** 1 Pediatric Cardiology Department, Fondazione CNR—Regione Toscana G. Monasterio, 54100 Massa, Italy; cantinotti@ftgm.it (M.C.); assanta@ftgm.it (N.A.); eliana.franchi@ftgm.it (E.F.); pitrino91@gmail.com (P.M.); 2 Institute of Clinical Physiology CNR-IFC, 56124 Pisa, Italy; 3 Department of Advanced Biomedical Sciences, Adult and Pediatric Cardiac Surgery, University of Naples Federico II, 80131 Naples, Italy; 4 Department of Pediatrics, Division of Pediatric Cardiology, Medical University Graz, 8036 Graz, Austria; martin.koestenberger@medunigraz.at; 5 Imaging Department, Fondazione CNR—Regione Toscana G. Monasterio, 54100 Massa, Italy; alberto.clemente@ftgm.it; 6 Taussig Heart Center, Department of Pediatrics, Johns Hopkins Hospital, Baltimore, MD 21205, USA; shelby.kutty@gmail.com; 7 Department of Medical Biotechnologies, Division of Cardiology, University of Siena, 53100 Siena, Italy; dascenzi2@unisi.it

**Keywords:** coronary artery origin, echocardiography, Athletes, Athlete’s heart

## Abstract

Anomalous aortic origin of coronary arteries (AAOCA) represents a rare congenital heart disease. However, this disease is the second most common cause of sudden cardiac death in apparently healthy athletes. The aim of this systematic review is to analyze the feasibility and the detection rate of AAOCA by echocardiography in children and adults. A literature search was performed within the National Library of Medicine using the following keywords: coronary artery origin anomalies and echocardiography; then, the search was redefined by adding the keywords: athletes, children, and high take-off. Nine echocardiographic studies investigating AAOCA and a total of 33,592 children and adults (age range: 12–49 years) were included in this review. Of these, 6599 were athletes (12–49 years). All studies demonstrated a high feasibility and accuracy of echocardiography for the evaluation of coronary arteries origin as well as their proximal tracts. However, some limitations exist: the incidence of AAOCA varied from 0.09% to 0.39% (up to 0.76%) and was lower than described in computed tomography series (0.3–1.8%). Furthermore, echocardiographic views for the evaluation of AAOCA and the definition of “minor” defects (e.g., high take-off coronary arteries) have not been standardized. An echocardiographic protocol to diagnose the high take-off of coronary arteries is proposed in this article. In conclusion, the screening of AAOCA by echocardiography is feasible and accurate when appropriate examinations are performed; however, specific acoustic windows and definitions of defects other than AAOCA need to be standardized to improve sensitivity and specificity.

## 1. Introduction

In recent years, the interest in the noninvasive evaluation of anomalous aortic origin of coronary arteries (AAOCA) has increased and multiple imaging, surgical and autoptic studies have been published [1,2,3,4,5,6,7,8,9,10,11,12,13,14,15,16,17,18,19,20,21,22,23]. AAOCAs are rare defects with estimates varying from 0.2% to 2.2% in autopsy series [23] and from 0.3% to 1.8% in Coronary Computed Tomography Angiography (CCTA) studies [4,7,24]. Despite being rare, AAOCAs have been recognized as important lesions with significant potential morbidity and mortality, since they represent the second cause of sudden cardiac death (SCD) in athletes [1,2]. Indeed, according to recent studies, AAOCAs account for 15.7% to 19% of all causes of SCD in young athletes [1,2,3]. Echocardiography is the first imaging modality to screen and diagnose coronary anomalies [3,4,5,6,7,8,9,10,11,12,13,14,15,19,20,21,22,23,24,25]. Recommendations for multimodality imaging of congenital coronary artery anomalies have been recently published (March 2020) [26], supporting the use of echocardiography as first-line imaging modality to detect AAOCAs. However, echocardiography may have limitations in the diagnosis of coronary defects due to technical issues such as interference by the ribs and lung, the high heart rate in neonates and children, and a poor acoustic window in some individuals [26]. Furthermore, the systematic assessment of AAOCAs during the screening for congenital heart disease is not yet universally performed [14,24,25,26,27,28,29,30,31,32]. While most of the articles [19,20,21,22,31], including guidelines [26], focused on major AAOCAs (e.g., mainly coronary artery origin from opposite sinus), the definition and clinical significance of some defects such as a high coronary artery take-off remain controversial [26,33]. Underestimated and often unknown high take-offs merit attention since these may be a cause of SCD in athletes [26,33]. 

The aim of the present manuscript is to systematically review the feasibility and the detection rate of echocardiography for the screening of AAOCA, with special attention paid to high take-off, and to highlight its strengths and limitations.

## 2. Methods

### Search Strategy 

The search strategy and study selection were carried out according to PRISMA guidelines. Potential publications were identified from a systematic search in the National Library of Medicine (PubMed access to MEDLINE citations; http://www.ncbi.nlm.nih.gov/PubMed/)/(accessed on 24 June 2020). The search strategy included a mix of medical subject headings and free text terms for the key concepts, starting from coronary artery origin anomalies and echocardiography. The search was further refined by adding the keywords athletes, children, and high take-off. In addition, we identified other potentially relevant publications using a manual search of references from all eligible studies and review articles, as well as from the Science Citation Index Expanded on the Web of Science. Two reviewers (M.C., M.K.) independently assessed all identified reports, and a consensus was reached for inclusion in the analysis. Titles and abstracts of all articles identified by the above search strategy were evaluated. Studies were excluded if they: (i) evaluated children with congenital heart defects (CHDs) that are often associated with coronary artery anomalies (e.g., transposition of the great arteries, tetralogy of Fallot), (ii) were focused on anomalous origin of the coronary artery from pulmonary artery (ALCAPA), Kawasaki or coronary artery fistula, (iii) used imaging modalities other than echocardiography (e.g., CT scan), and (iv) were written in languages other than English. 

## 3. Results

### 3.1. Literature Search and Demographic Characteristics

Fifty-six articles were identified in the search for potential inclusion in the study. Thirty-five studies were excluded based on the criteria listed above: 9 evaluated children with CHD; 25 were focused on ALCAPA, Kawasaki or fistula; 10 used CT or Cardiac Magnetic Resonance (CMR) imaging; 3 were written in languages other than English. Nine articles were included in the final analysis [13,15,19,20,21,22,23,24,31], for a total of 33,592 children and adults (age range: 12–49 years) where AAOCAs have been systematically screened by echocardiography (Table 1). Six studies had prospective designs [13,19,20,21,24,31], while three were retrospective [15,22,23]. Six studies were conducted on 6599 young and adult athletes (age range: 12–49 years) [13,15,19,20,24,31], while three were on the general population [20,22,23]. Two studies were conducted on elite athletes [13,19], while the remaining [15,18,20,24,31] used athletes of different sports at different levels. (Table A1 in Appendix A).

### 3.2. Feasibility of Visualizing Coronary Arteries’ Origins

A good feasibility of visualizing origin of coronary arteries was described by most of the authors [15,19,21,24,31], including studies published in the 1990s [19,20]; in three studies, the feasibility was not reported [13,22,23]. Overall, the feasibility of coronary artery origin visualization varied from to 90% [20] to 98.5% [21] and was higher in children compared to adults [21]—see Table 2. The correct visualization of the left common artery (LCA) origin was considered feasible for 98% [15] to 100% [31] of the subjects, while the feasibility for the visualization of right coronary artery (RCA) origin varied from 80% [19] to 96% [24]. When the visualization refers to the proximal course of the coronary arteries, the feasibility was lower, ranging from 81–82% [15] to 98.5% [21].

### 3.3. Echocardiographic Views Employed to Visualize Origin of Coronary Artery

The origin of coronary arteries was assessed in the parasternal short-axis view by all the authors [13,19,20,21,22,23,24,31], which is well in accordance with recent guidelines [26]. Parasternal long-axis [13,23] or para-sagittal planes [23] views, also recommended [26], were employed only by two authors. Only two studies [21,22] used the color Doppler with reduced color gain (e.g., 15–40 cm/s) to detect coronary flow. Thus, the use of specific protocols for the evaluation of coronary artery origin may increase the detection rate of anomalies; indeed, Thankvel and colleagues [22] reviewed their experience before and after the introduction of a new screening method for the evaluation of AAOCA that extends the parasternal short-axis view into the ascending aorta in children and adolescents. They found that the detection rate of AAOCA improved from 0.02% (in 5669 subjects screened) to 0.22% (in 6428 subjects screened).

### 3.4. Detection Rate of Major and Minor AAOCAs by Echocardiography

**Figure 1:**
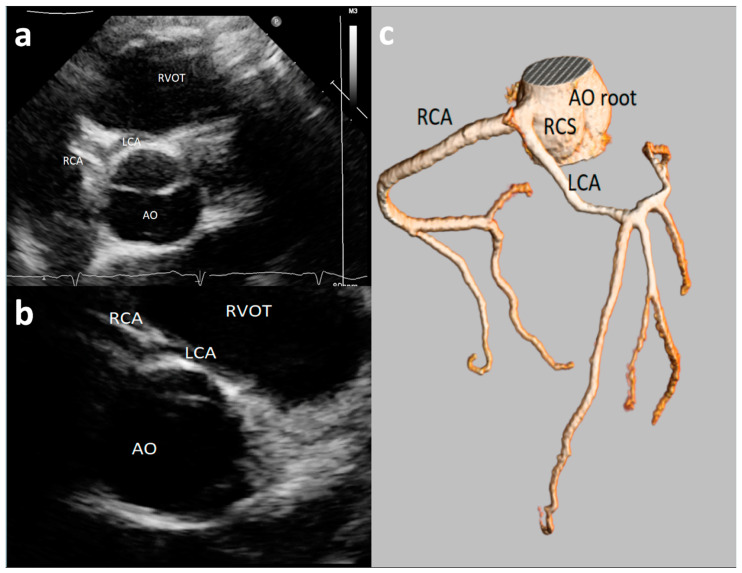
An asymptomatic 14-year-old boy evaluated with negative stress test for murmur at routine soccer screening. Origin of the left common artery (LCA) from the right sinus of Valsalva was incidentally discovered at echocardiography ((**a**,**b**)—short-axis view with slightly different angulation) and confirmed by Coronary Computed Tomography Angiography (CCTA) (**c**). RCA = right coronary artery; LCA = left common artery; RCS = right coronary sinus; AO = aortic root; RVOT = right ventricle outflow tract.

Major AAOCAs were usually defined as RCA and LCA origins from opposite wrong sinus (Figure 1), a single ostium coronary artery, or left circumflex artery (CFx) originating from right coronary sinus [14,15,20,23,24,31]. The detection rate of major AAOCAs greatly varied among the different studies, from 0.0% [15,24,31] to 0.09% [20] and up to 0.76% [23]. The positive predictive value of echocardiography (with confirmation at either coronary angiography or CT angiography) in the diagnosis of major AAOCAs was high, varying from 87.5% [21] to 100% [20].

**Figure 2:**
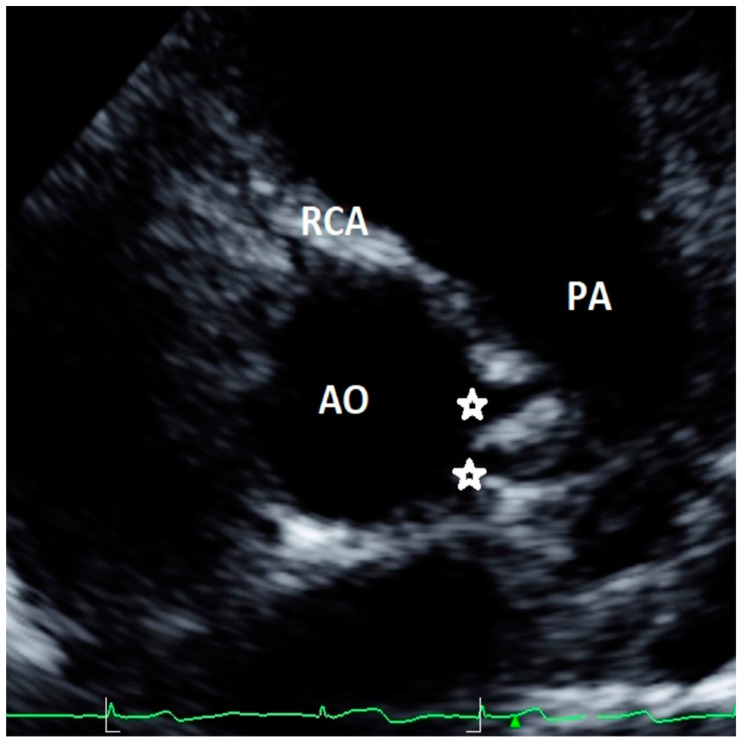
Asymptomatic 13-year-old boy evaluated with negative stress testing for murmur at preparticipation evaluation for sports competition. Parasternal short-axis view at the level of aortic root showing separate origins of circumflex (CFx) and left descending artery (LAD).

Minor AAOCAs were described for 1.5% [13] to 2.6% [19] of the cases: they included separate ostia for left anterior descending artery and CFx from left sinus, two distinct ostia in the right sinus for RCA and the conus branch, and a short left main coronary artery—<5 mm—or small fistulas. A case of minor AAOCA from our case series, with two distinct ostia originating from the left sinus, is shown in Figure 2. However, definition and clinical significance of minor and major AAOCAs varied among the studies. Indeed, the study by Lytrivi and colleagues [23], reporting the highest number of AAOCAs (111 patients out of a cohort of 14,546 subjects), did not distinguish between major and minor defects. Gerling et al. included into low-risk AAOCAs also high take-off of coronary arteries with acute angle that may be at risk of SCD [33]. Four studies did not evaluate minor AAOCAs [15,22,24,31]. 

### 3.5. Symptoms, ECG, Stress Testing, and Clinical Management

Information about clinical data, including the indication to echocardiography, is reported in only four studies [13,20,21,23] (Table A1 in Appendix A). Most of the examinations were just screenings [13,20,21,23], while in a limited case there was a clinical indication. Among the 59 AAOCAs evaluated in these studies, only 10 patients presented symptoms (chest pain and/or dyspnea) and one case was resuscitated after cardiac arrest related to ventricular fibrillation; in this case, a major AAOCA was found, with LCA originating from the wrong sinus (e.g., the right sinus of Valsalva). The basal electrocardiogram was completely normal [13,20] or showed no specific defects [21,23], such as left or right ventricular hypertrophy, T wave inversion or St depression in V5–V6, left-axis deviation [21,23] in these symptomatic subjects. A stress test was performed only in 23 out of 59 AAOCAs subjects, being positive only in five cases [13,20,21]. Myocardial scintigraphy was employed as stress testing modality and resulted positive in 4 out of 9 cases [20,21]. Unroofing surgery was performed in 10 cases [21,23] (including two with positive scintigraphy, one urgent case and seven cases where results of stress tests are not available), while two authors reported indication for sport eligibility [13,20]. Athletes with major AAOCAs were disqualified [20], while athletes with high RCA take-off did not undergo sports restriction [13]. 

### 3.6. Coronary Artery High Take-Off

**Figure 3:**
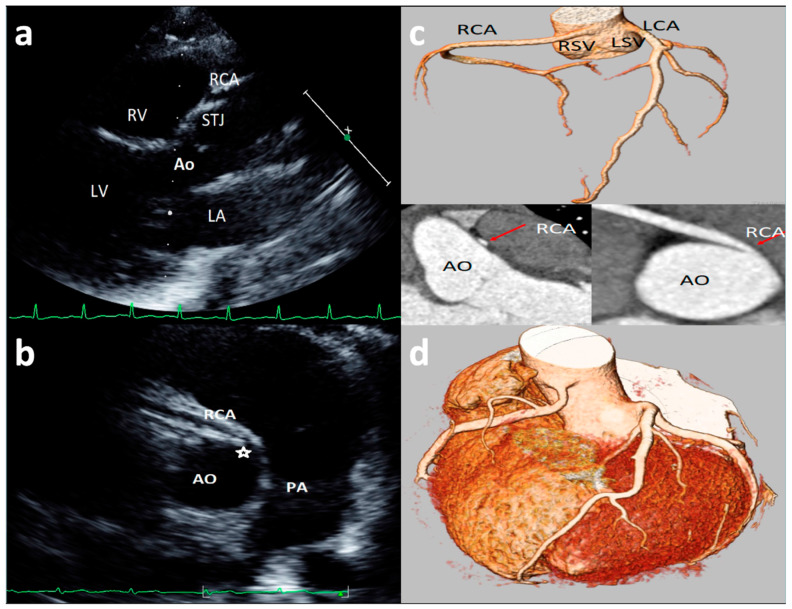
Asymptomatic 17-year-old boy evaluated for the presence of two premature ventricular beats at peak of stress test in routine preparticipation evaluation for sports competition. Parasternal long-axis view showing right coronary artery (RCA) high take-off coronary artery above the sinus of Valsalva (**a**). The acute angulation origin is clearly visualized in short-axis view (**b**) and was confirmed at CT (**c**). After CT confirmation of the defect, a myocardial scintigraphy was performed revealing a small ischemia (8%) in the RCA territory at peak. We advised sport restriction. Ao = aorta, LCA = left common coronary artery, RCA = right coronary artery, LSV = left sinus of Valsalva, RSV = right sinus of Valsalva. (**d**) is an example of 73-year-old man showing symptoms of dyspnea. 3D-Volume Rendering CCTA showing the right dominant coronary artery (RCA) high take-off two centimeters distal to the sinutubular junction at the level of the anterior ascending aortic wall.

High take-off coronary artery is a rare anomaly [33,34,35,36,37,38,39,40,41,42,43] that may present in isolation or associated with other congenital cardiac malformations [35,36,37,38,39,40,41,42,43], mainly identified for the RCAs (up to 84.46% of cases) [33]. There is still limited literature on the visualization and definition of RCA high take-off by echocardiography [13,14,23]; although there is not a consensus on the definition of high take-off by echocardiography, all the studies included in the present review defined high take-off as an origin above or distal to the sinutubular junction (STJ) [13,14,23]. An example of high take-off of RCA from our case series is reported in Figure 3. A very recent [13] study, of 1045 consecutive elite adolescent football players, identified coronary high take-off origin in 13 subjects (i.e., 1.14%). Eccentric RCA origin with a high take-off and partial intra-arterial course was observed in two cases (with no slit-like ostium and no intramural course); high take-off origin of the RCA with acute angle was observed in one case; high take-off of the RCA origin (with no intramural or slit-like orifice) was observed in 11 cases. Of the latter 11 cases, diagnosis was feasible only from the parasternal long-axis view, where the ostium of the RCA was measured from 2.3 to 6.8 mm above the sinutubular junction [13]. Lytrivi et al. [24] documented RCA high take-off in 53 cases (0.36%), LCA high take-off in four cases, and high take-off of both the coronary arteries in two cases of a valuable cohort of 14,546 pediatric subjects [23].

There is relatively limited literature on high take-off of LCA [26], and echocardiographic reports are extremely limited [14,23]. Only one study among the nine included in this review reported four cases of high LCA. Of these, a normal intracardiac anatomy was found in one case and associated defects (including one ventricular septal defect, one patent arterial duct, one aortic coarctation) were found in three cases [23]. No clear indications for the appropriate acoustic window to be used for evaluating the LCA take-off were reported in this study. 

## 4. Discussion

In the present article, we reviewed all studies that systemically evaluated AAOCAs by transthoracic echocardiography [13,15,19,20,21,22,23,24,31]. Our analysis reveals a particularly good feasibility to visualize coronary artery origin and proximal course by transthoracic echocardiography. Despite this extremely high feasibility, the detection rate of AAOCA by echocardiography seems to be suboptimal. Indeed, the known estimates of AAOCA by CCTA, CMR, and autoptic studies range from 0.3% to 1.8% [4,7,23,34,35,36,37,38,39,40,41,42,43,44,45,46], while echocardiographic studies reported significant lower incidences, ranging from 0% [15,24,30] to 0.09% [20] and up to 0.76% [23]. However, echocardiographic studies with low incidences of AAOCAs were either retrospective [15] or used echocardiography in a fast-echo approach in the context of preparticipation evaluation of athletes [24,31]. Coronary artery origin was consistently evaluated in a parasternal short-axis view by all the authors [13,15,19,20,21,22,23,24,31], in agreement with the recent recommendations for multimodality assessment of congenital artery anomalies [26]. However, when protocols that systematically analyzed coronary arteries origin in different planes (e.g., parasternal, parasagittal planes, short-axis view extended to the ascending aorta) were adopted [13,22,23], the detection rates of AAOCAs were significantly higher [21,22,23]. 

**Figure 4:**
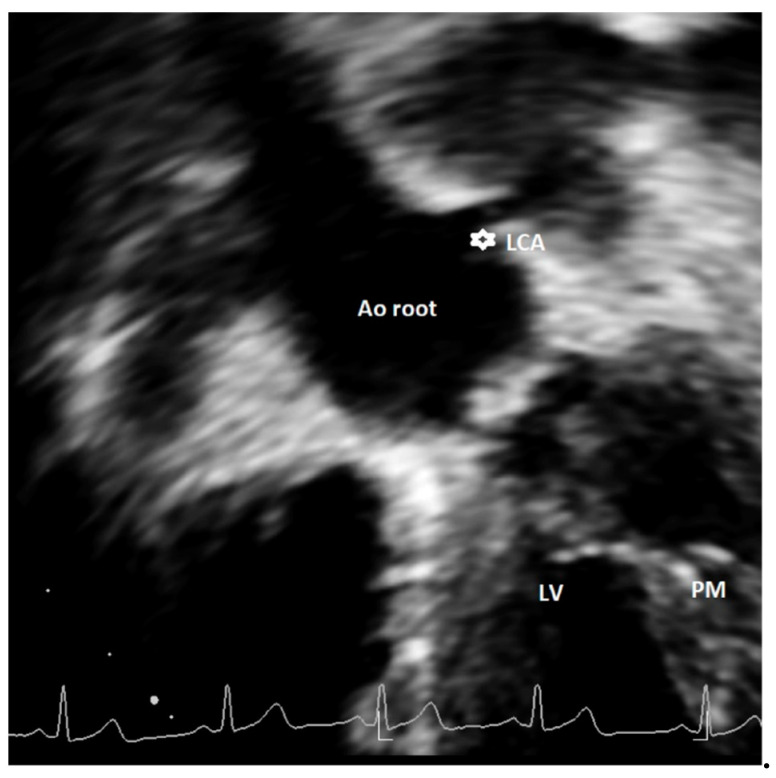
Modified apical 5-chamber view showing high take-off of left coronary artery (asterisk). This is a case of an asymptomatic 14-year-old female, with incidental diagnosis of bicuspid aortic valve. Ao = aorta, LCA = left common coronary artery, LV = left ventricle; PM = papillary muscle.

Discrepancies exist in the definition of major and minor AAOCAs, especially regarding the classification of a high take-off of coronary artery origin. Unfortunately, limited echocardiographic data are currently available in the literature [13,14,23]. Furthermore, most of the CT studies adopted a different definition as compared to echocardiographic articles. Indeed, in most cases, CT studies defined high take-off as a height >1 cm or >20% the depth of the sinus above STJ [26], a height >0.25 cm above the sinutubular junction, and a minority any height above the STJ [26]. Echocardiographic studies all used the latter definition, identifying the high take-off as an origin above or distal to the STJ [13,14,23]. The use of different cut-offs to define a high take-off has the consequence of affecting prevalence [33]; indeed, in CT studies where the definition of a height >1 cm or >20% the depth of the sinus above sinutubular junction was used, the incidence of RCA high take-off was 0.202%. Conversely, in those that employed a height >0.25 cm above the sinutubular junction as a definition, it decreased to 0.199% [33]. When the high take-off of RCA was defined as any height of origin above STJ, the prevalence of this defect increased [33] up to 0.364% [26]. In echocardiographic studies that used the latter definition, the reported incidence of RCA high take-off was even higher than in CT studies, ranging from 0.36% [23] to 1.14% [13]. This definition may overestimate the prevalence of high take-off but, most importantly, it may have the consequence to classify benign variants as malignant anomalies potentially at risk of SCD [33]. As recently reviewed [26], 3 of 12,899 (0.023%) cases of high take-off coronaries that originated more than one centimeter above the sinutubular junction were associated with SCD. Notably, although preferable in adults, the use of fixed criteria (such as 1 cm above the sinutubular junction) may have relevant limitations in children where aortic dimensions are smaller than in adults; therefore, some authors [44] proposed the adoption of relative criteria, such as coronary orifices that arise 120% or more of the depth of the sinus of Valsalva or 20% or more the depth of the sinus above the STJ. A comprehensive evaluation of a high take-off of coronary arteries should also include other important characteristics, such as the presence of slit-like ostium, stenosis, the interarterial course, and intramural course [26,33,34,35]. Notably, a high take-off associated with acute angulation from the aorta, an intramural or an interarterial course comprising 4% of the defects, are more at risk for the development of SCD [26]. This specific characteristic may be studied by echocardiography [13], but this imaging technique has inherent limitations for an accurate definition of these characteristics that need a comprehensive multimodality approach for their definition, as suggested by the current recommendations [26]. Furthermore, based on previously published studies, no clear indications exist for the appropriate acoustic window to be used for other coronary anomalies, such as the high take-off of coronary arteries, and particularly for LCA. Current recommendations [26] suggest using a coronal subcostal view to visualize LCA origin; however this window can be easily utilized only in neonates and children. We propose in this article an additional acoustic window that may allow for evaluation of high take-off of LCA by using a modified apical five-chamber view (see Figure 4). So far, we have tested this projection in a limited number of cases with suspicion of LCA high take-off or when origin of LCA was not seen by conventional short-axis and parasternal long-axis views, with encouraging results. 

The clinical management of patients with AAOCAs is challenging, particularly in asymptomatic subjects practicing sport [44,47,48,49,50], and discrepant approaches (e.g., surgery vs. follow-up with or without exercise restriction) [49,50,51,52] have been adopted so far. Indeed, athletes with AAOCAs are often asymptomatic [20,21] or with mild symptoms (e.g., chest discomfort, palpitations), and stress testing can be frequently normal (or with minor abnormalities) [9,44]. Mery et al. [47] proposed a protocol where only patients with symptoms ascribed to ischemia (aborted SCD, syncope during or following exercise), or asymptomatic high risk anatomy (intramural, abnormal ostium) or with established perfusion defects undergo cardiac surgery; otherwise, no intervention neither exercise restriction is recommended. 

Current American Heart Association/American College of Cardiology (AHA/ACC) guidelines [52] suggest that, in asymptomatic athletes with a coronary artery originating from the wrong sinus of Valsalva and negative stress test, permission to compete can be considered after adequate counseling (Class IIa; Level of Evidence C) [52]. However, when the artery passes between the pulmonary artery and aorta, athletes should be restricted from participation in all competitive sports, except for Class IA sports, before surgical repair, independently from the presence of symptoms (Class III; Level of Evidence B) [52]. If athletes with AAOCA exhibit symptoms, arrhythmias, or signs of ischemia on exercise stress test, they should be restricted from participation in all competitive sports, except for Class IA sports, before a surgical repair (Class III; Level of Evidence C) [52]. The Italian guidelines [53] suggest restricting participation in competitive sports in case of a coronary artery originating from the wrong sinus of Valsalva; conversely, in case of an anomalous origin of the left circumflex coronary (CFx) from the right sinus of Valsalva and demonstrated absence of myocardial ischemia, competitive sports are permitted with a tailored decision with a case-by-case approach [53]. 

## 5. Limitations

Our research was focused on 2D transthoracic echocardiography; therefore, studies evaluating the origin of coronary arteries by three-dimensional and/or transesophageal echocardiography were not included in the present review. There has been limited application [54] of 3D echocardiography for the screening of coronary arteries. Despite 3D echocardiography potentially allowing a better visualization of coronary anomalies in a good acoustic window, 2D echocardiography has greater sensitivity [54].

Studies included in this review were limited, heterogeneous, made use of different technology and expertise, and had differences in terms of study populations. Therefore, data were too heterogeneous to perform a valid meta-analysis. However, interesting data on the feasibility and accuracy of the echocardiographic analysis in this field can be collected and interpreted and may represent food for thought. Data for some defects (particularly a high take-off of LCA) are too limited to draw any type of conclusion regarding the feasibility and the accuracy of echocardiography for diagnosis. Additionally, the projection we propose for LCA high take-off visualization (e.g., modified apical five-chamber view) needs to be validated in wider, prospective studies. Furthermore, some defects (such as myocardial bridge, intramural course) cannot be easily visualized by echocardiography and represent a limitation of transthoracic echocardiographic for the estimation of coronary anomalies in terms of coronary course in the myocardium. 

## 6. Conclusions

Echocardiographic evaluation of the origins of coronary arteries by transthoracic echocardiography is feasible and accurate. The use of systematic protocols including different acoustic windows in addition to a basic short-axis view (e.g., parasternal, parasagittal views, short axis extended into the ascend ending aorta) is essential for the optimization of imaging for congenital coronary artery anomalies and for improving their detection by echocardiography which, at present, remains suboptimal compared to other imaging modalities such as CCTA or CMR. The definitions of some anomalies (such as a high take-off) need to be standardized and the clinical significance of AAOCAs should be clarified by further research. Since data on LCA high take-off assessed by echocardiography are extremely limited, a method to visualize this defect has been proposed in the present article.

## 7. Perspectives

Since abnormal origins of coronary arteries are the second most common cause of sudden cardiac death in apparently healthy athletes [1,2,3,55], the echocardiographic evaluation of origin and proximal course of coronary arteries should be a fundamental part in screening of an athlete. However, the definitions of some coronary artery origin anomalies (such as a high take-off) and their clinical significance have not been completely defined yet. There is a need for recommendations for the definition and the clinical risk classification of AAOCAs as well as for the decision making, including sports eligibility or disqualification. In fact, although concealed life-threatening abnormalities of the coronary arteries should be diagnosed, some defects are benign and it is important to avoid creating unjustified anxiety, using invasive and expensive examinations, or indicating sport restriction when functionally benign defects are demonstrated. 

## Figures and Tables

**Table 1:** Methodology of studies who systematically screened coronary artery origin by echocardiography.

Study, Authors	Study Design	Echo Protocol	Coronary Artery Echo View	Echo Machine	High Take-off Definition
Hyot [15] 2017, USA	Retrospective Athletes Different sports, different levels, pre-participation screening	NR	NR	NR	-
Zeppilli [20] 1998, Italy	Prospective Athletes Different sports, different levels, preparticipation screening	NR	PSA	Sonoline CD (Siemens, Medical Solutions USA, Inc, Mountain View, CA, USA)	-
Pellicia [19] 1993, Italy	Prospective Athletes Elite, different sport, preparticipation screening	NR	PSA	NR	-
Gerling [13] 2018, Germany	Prospective Athletes Adolescent elite football players-participation screening	NR	PSA or PLA	Aplio 500 CV (Toshiba Medical System, Otawara, Japan), Vivid I, (General Electric, Healthcare, USA)	High take-off above to the SJ
Lombardara [21] 2013, France	Prospective General population	Standard full examination	Modified PSA Color doppler with decreased velocity	IE33 Philips Healthcare (DA best, The Netherlands)	-
Thanraval [22] 2014, USA	Retrospective General pediatric population	NR	2010 2D and color doppler PSA at the level of coronary sinuses 2011 Added PSA superiorly into the Asc Ao +/-color Doppler with decreased velocity	Siemens Sequoia C512 ultrasound equipment (Siemens, Medical Solutions USA, Inc, Mountain View, CA, USA) or Philips iE33 (Philips Medical Systems, Bothell, WA, USA)	-
Lytrivi [23], 2008, USA	Retrospective General pediatric population	NR	PSA, left or right PLA and/or parasagittal planes	Aplio 500 CV (Toshiba Medical System, Otawara, Japan)	High take-off distal to the SJ
Wyman [24] 2007, USA	Prospective Athletes Different sports, different levels, preparticipation screening	5 minutes echo	PSA	Siemens Acuson Sequoia, General Electric Vivid 7, USA. Philips Sonos 5500 (Philips Medical Systems, Bothell, WA, USA)	-
Maron [30] 1987, USA	Prospective Athletes Adolescents, different sports, different levels, preparticipation screening	Standard full examination	LMCA origin PSA	Advance Technology Laboratory (ATL) Mark 500 (Advanced Technology Laboratory-*Seattle*)	-

NR = not reported; Asc A0 = ascending aorta; PSA = parasternal short-axis view, PLA = parasternal long-axis view, LMCA = left main coronary artery, SJ = sinutubular junction.

**Table 2:** Feasibility of coronary artery origin visualization by echocardiography and diagnosis of anomalies.

First Author, Year of Publication	Sample Size and Age	LCA	RCA	All	Major Anomalies	Minor Anomalies
Hyot [15], 2017, USA	146 (18–23 years)	98% 81%	98% 82%	NR	0%	NR
Zeppilli [20], 1998, Italy	3650 (30 ± 12 years)	NR	NR	Ostium and proximal tract: 90%	0.09%: RCA from left sinus, LCA from right sinus	1.6%: separate origin of LCA and CFx from left sinus of Valsalva and two distinct ostia in the right sinus for RCA and the conus branch
Pellicia [19], 1993, Italy	1273 (13–49 years)	98.7%	80%	93%	0%	2.19%: Separate ostia for LCA and CFx (n°6), short LCA (<5 mm) with real bifurcation (n°22)
Gerling [13], 2018, Germany	1045 (12–15 years)	NR	NR	NR	0.19% RCA with high take-off and partial intra-arterial course (n°2)	1.5% RCA high take-off with acute angle (n°1), small fistulas (n°2), ectasias of LCA (n°2), and RCA high take-off (n°11) Incidence of High take-off 1.14%
Labombarda, [21], 2013, France	350 (84% adult,16% children)	NR	NR	Ostium and first tract 98.5% children	0.39% RCA from left sinus (n°8), single coronary osmium (n°3), LCA from right sinus (n°1), fistula (n°1)	NR
Thanraval [22], 2014, USA	2010 (n 5669 0–21 years) 2012 N 6.428 (0–21 years)	NR	NR	NR	0.02% Intramural LCA for right sinus 0.22% RCA from left sinus (n°13), LCA from right sinus (n°2)	NR
Lytrivi [23], 2008, USA	14,546	NR	NR	NR	0.76% Major and Minor RCA from LSV (n°24), Cx from RCA (n°9), Single RCA (n°8), LCA from RSV (n°6), LAD off RSV (n°3), Dual LAD (n°1), Single LCA (n°1), RCA high take-off (n°53) (0.36%, LCA high take-off (n°4), high take-off of both (n°2), fistulas (n°57) Incidence of RCA high take-off 0.36% All 0.41%	NR
Maron [30], 1987, USA	90 17–30 years	100%	NR	NR	0%	
Wyman [24], 2008, USA	395 17–23 years	99%	96	NR	0%	

LAD = left anterior descending artery; LCA = left common coronary artery, NR = not reported, RCA = right coronary artery, CFx = circumflex artery, LSV = left sinus of Valsalva, RSV = left sinus of Valsalva.

## Data Availability

The datasets used for the current study are available from the corresponding authors upon reasonable request.

## Appendix A

**Table A1:** Studies reporting information about the reason of echocardiographic examination, ECG, stress test results and management.

Author	Sample Size and Age	Anomaly/Course	Reason for Referral	ECG	Stress Test	Management
Zeppilli [20], 1998, Italy	3,650 (30 ± 12 years) Athletes		RCA from LSV (n°2):1 screening, 1 fatigue during effort: LCA from RSV (n°1): LBB at stress test	All normal	RCA from LSV positive and 1 negative myocardial perfusion scintigraphy. Positive had left-axis deviation at ECG stress test. 1 LCA from RSV: positive scintigraphy	No (old study) All disqualified from competition
Gerling [13], 2018, Germany	1,045 (12-15 years) Athletes	RCA high take-off (n°14), interarterial course (n°2)	Screening all Asymptomatic	All negative	All negative stress test	No sport restriction
Labombarda, [21], 2013, France	350 (84% adult,16% children) Athletes	LCA from RSV; interarterial (n°1) RCA from RSV; interarterial (n°8)	LCA from RSV, Cardiac arrest (n°1) SC (n°3), 2 acute coronary syndrome, 1 asympt. RCA from LSV (n°8) 4 asympt, 1 stroke, 1chest pain, 2 dyspnea	LCA from RSV (n°1): Normal SC (n°3):1 T wave inversion, 2 normal RCA from LSV (n°8): 3 normal, 1 LVH, T wave inversion (V5, V6), 1 Q wave (V2, V3),1 ST changes (V5-v6),1 AF	LCA from RSV A (n°1): not done (urgency) SC (n°3): 1 positive (myocardial perfusion scintigraphy), 1 normal, 1 not done. RCA from LSV (n°8): 1 positive (myocardial perfusion scintigraphy, 3 negatives (scintigraphy), 4 not done	Surgery: 1 LCA from RSV, 1 SC,3 ARCA Conservative management: all the rest. No indication on sport
Lytrivi [23], 2008, USA	14546 General pediatric population	LCA from RSV (n°6) 3 interarterial, 1 retro-aortic, 1 LAD anterior, Cx posterior RCA from LSV (n°24) 1 intramural, 1 anterior, 22 interarterial	LCA from RSV (n°6): 1 chest pain, 1 murmur, 2VSD, 2 pre-liver tx RCA from LSV (n°24): 2 chest pain, 2 tachycardia, 3 suspected coronary anomalies on echo, 2 VSD, 2 PDA. 1 AS, 1 ASD, 1 Down syndrome, 3 murmur, 1 abnormal ECG, 1 metabolic syndrome	LCA from RSV (n°6): 3 normal, 1 LVH, 2 RVH RCA from LSV (n°24): 17 normal. 2 RVH, 2 BVH, 1 left-axis deviation, 1 superior axis		Surgery LCA from RSV (n°3): 1 unroofing, 2 surgery for VSD RCA (n°8): from LSV, 4 unroofing, 4 surgery for VDS/ASD Conservative management: all the rest. No indication on sport

AF = atrial fibrillation, AS = atrial stenosis, asympt = asymptomatic, ASD = atrial septal defect, Cx = circumflex artery, ECG = electrocardiogram, LAD = left anterior descending, LVH = left ventricular hypertrophy, LSV = left sinus of Valsalva, PDA = patent arterial duct, RCA = right coronary artery, RSV = right sinus of Valsalva, RVH = right ventricular hypertrophy, SC = single coronary artery ostium, tx = transplant, VSD = ventricular septal defect.
